# Supplementary material for: Transcriptome and metabolome analyses reveal regulatory networks associated with nutrition synthesis in sorghum seeds
Source: Commun Biol. 2024 Jul 10;7:841. doi: 10.1038/s42003-024-06525-7 (PMC11237005; doi:10.1038/s42003-024-06525-7)
Supplement: Supplementary file 2 — Supplementary Information [file 42003_2024_6525_MOESM2_ESM.docx]

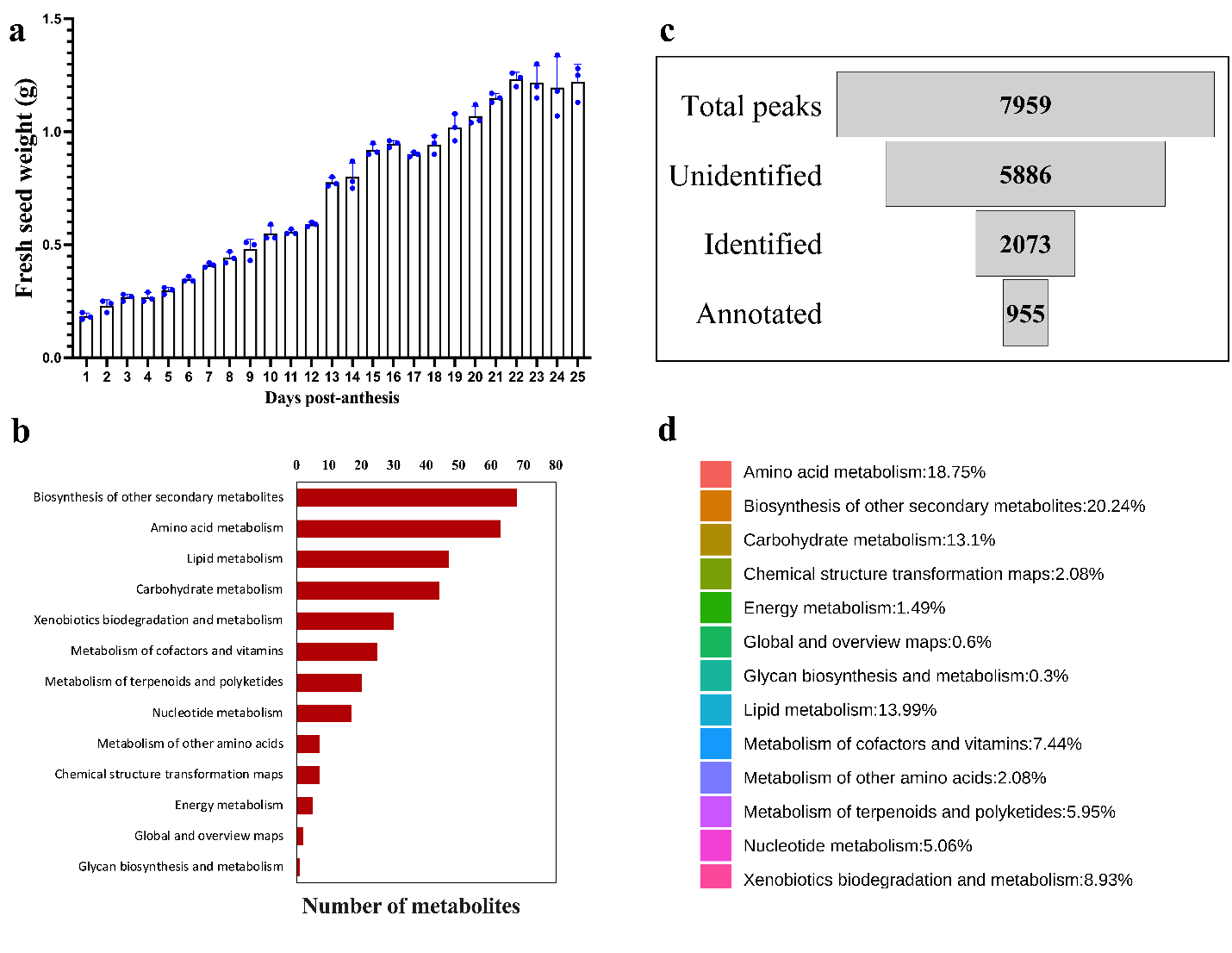
**Supplementary Figures**

**Supplementary Fig. 1:** (a) Changes in seed fresh weight after fertilization to 25 dpa. (b) Overview of metabolome data. (c) Classification of differential metabolites into KEGG metabolic pathways. (d) Functional classification of annotated metabolites.


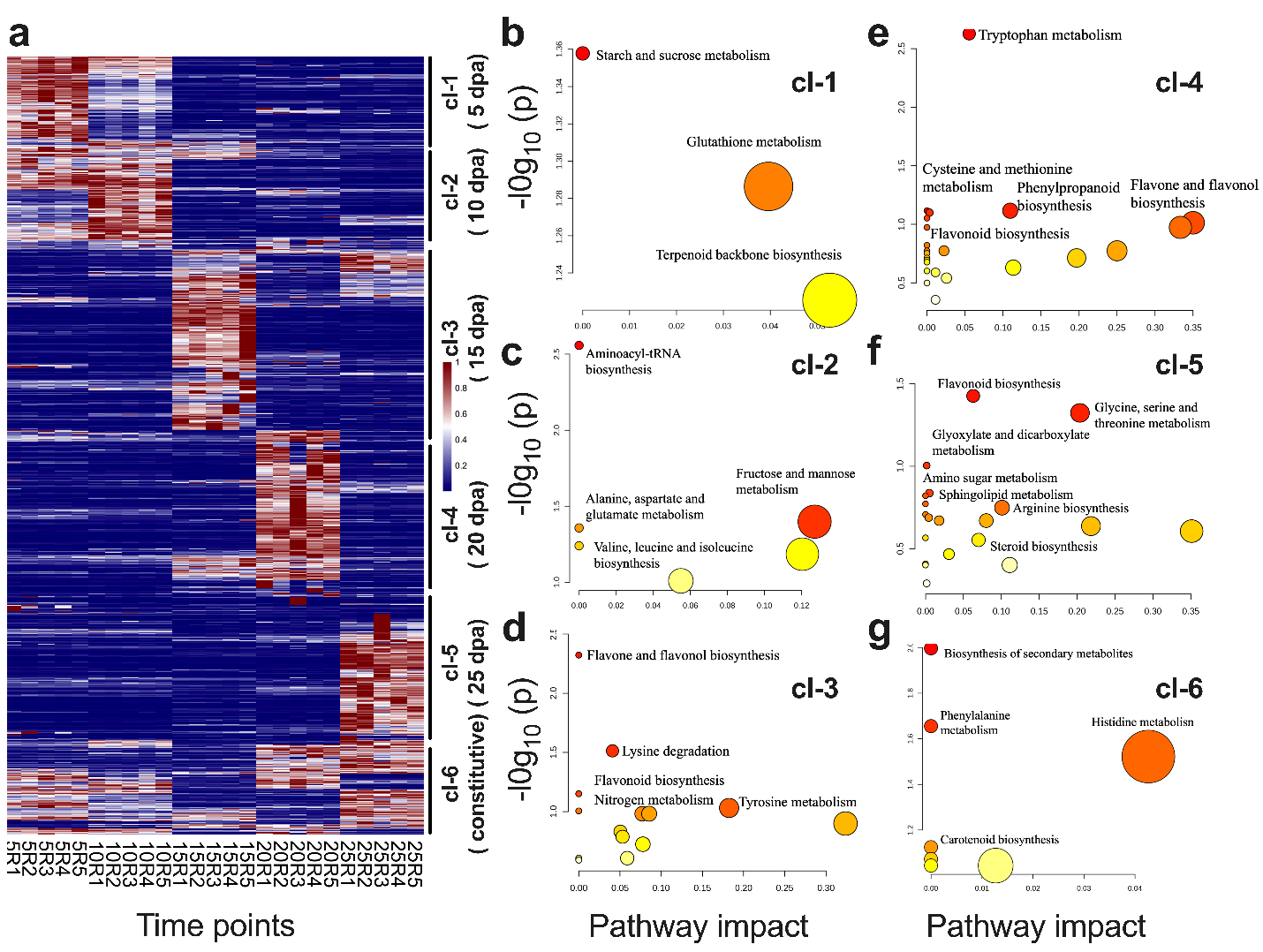


**Supplementary Fig. 2:** (a) Heatmap showing metabolite trends in five key timepoints of seed development (5, 10, 15, 20, and 25 dpa). (b–g) KEGG metabolic pathway categorization of the metabolites present in each cluster, which reflects enriched metabolites for a specific stage. Node colors represent P-values, with white and red indicating lower and higher P-values, respectively. Node radii correspond to pathway impact values, with smaller and larger radii indicating lower and higher impact values, respectively.


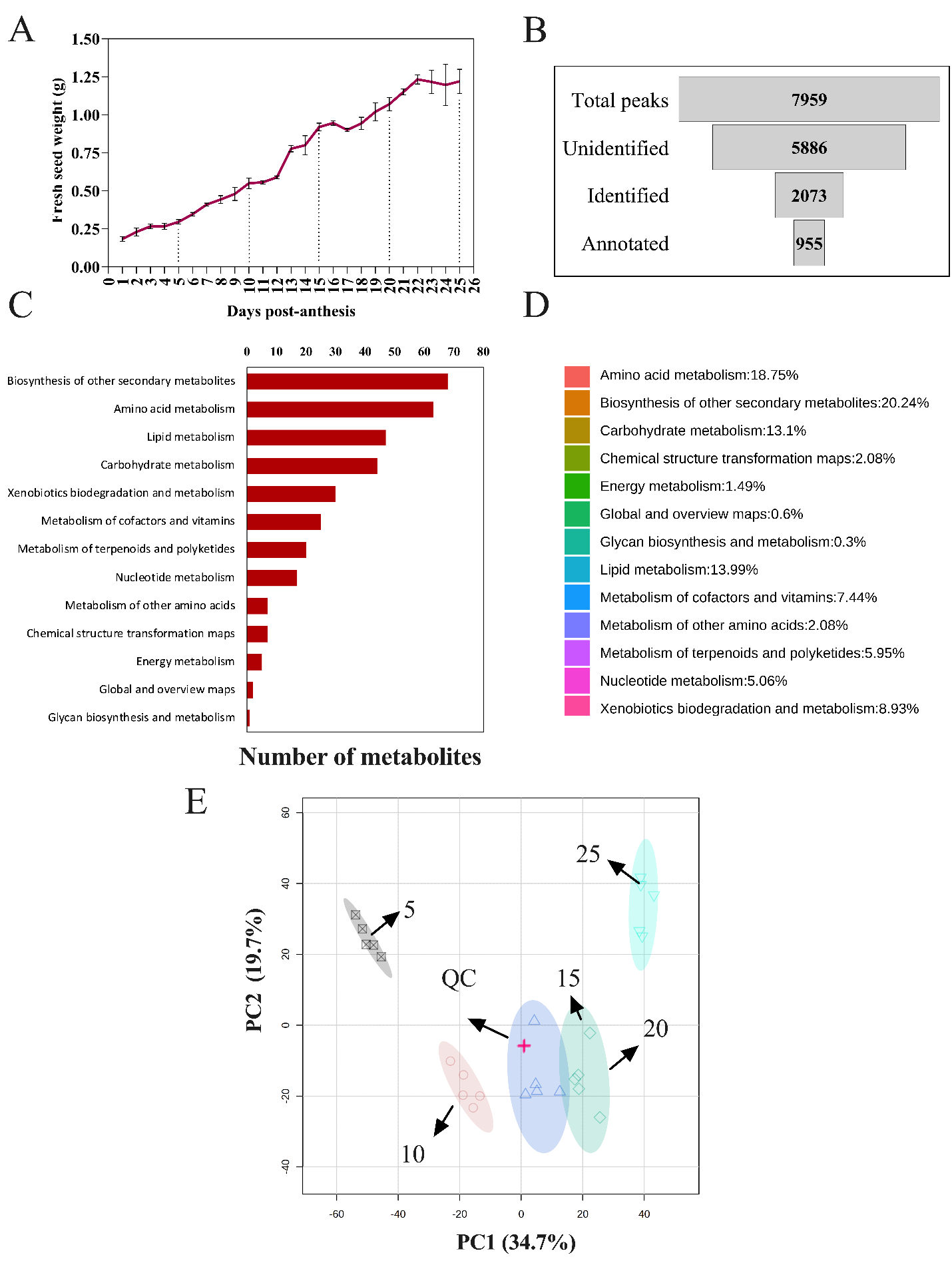


**Supplementary Fig. 3:** Principal component analysis of metabolomic data using 2071 putatively identified metabolites in sorghum seeds at the five timepoints. QC reflects quality control samples.


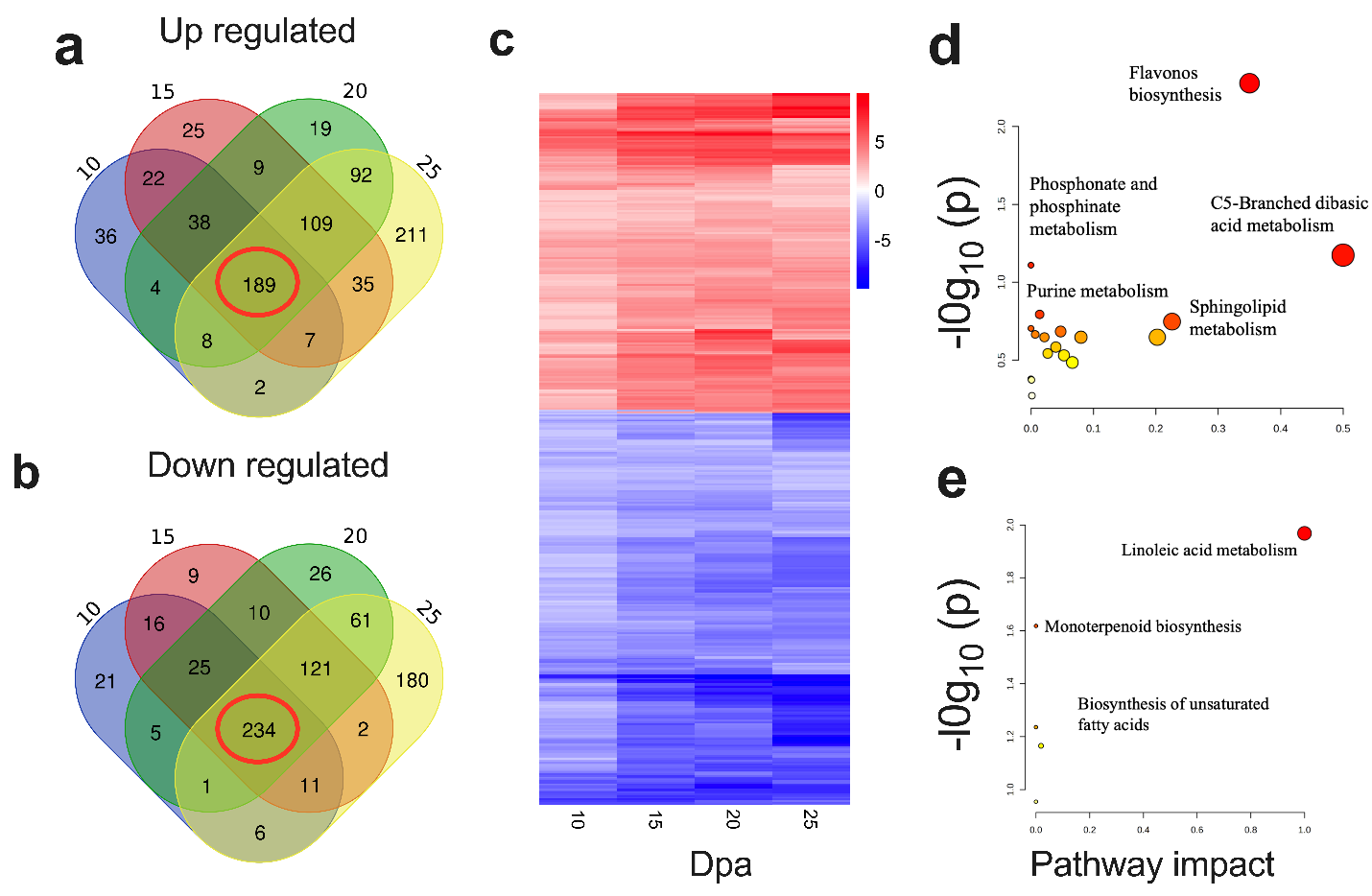


**Supplementary Fig. 4:** (a & b) Venn diagrams displaying the overlap of upregulated (a) and downregulated (b) differential metabolites among the investigated comparisons (5 vs. 10, 15, 20 and 25 dpa). (c) Heatmap showing metabolite trends (red: upregulated; blue: downregulated) in aforementioned comparisons. (d-e) KEGG metabolic pathway categorization of the upregulated and downregulated metabolites. Node colors represent P-values, with white and red indicating lower and higher P-values, respectively. Node radii correspond to pathway impact values, with smaller and larger radii indicating lower and higher impact values, respectively.


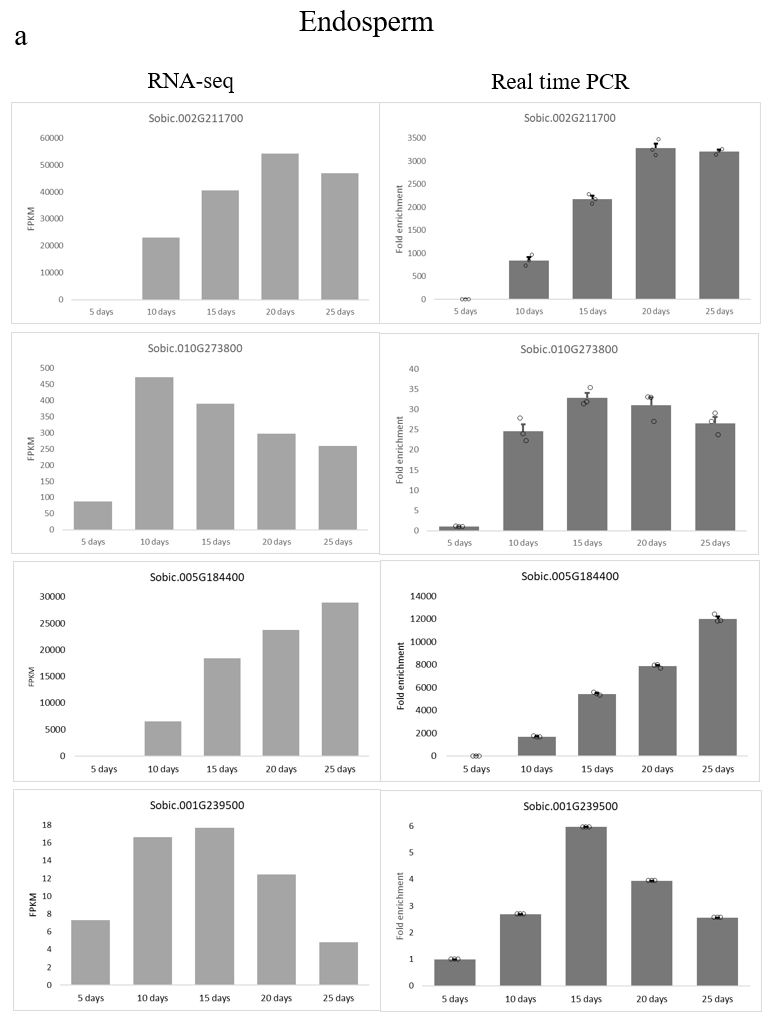


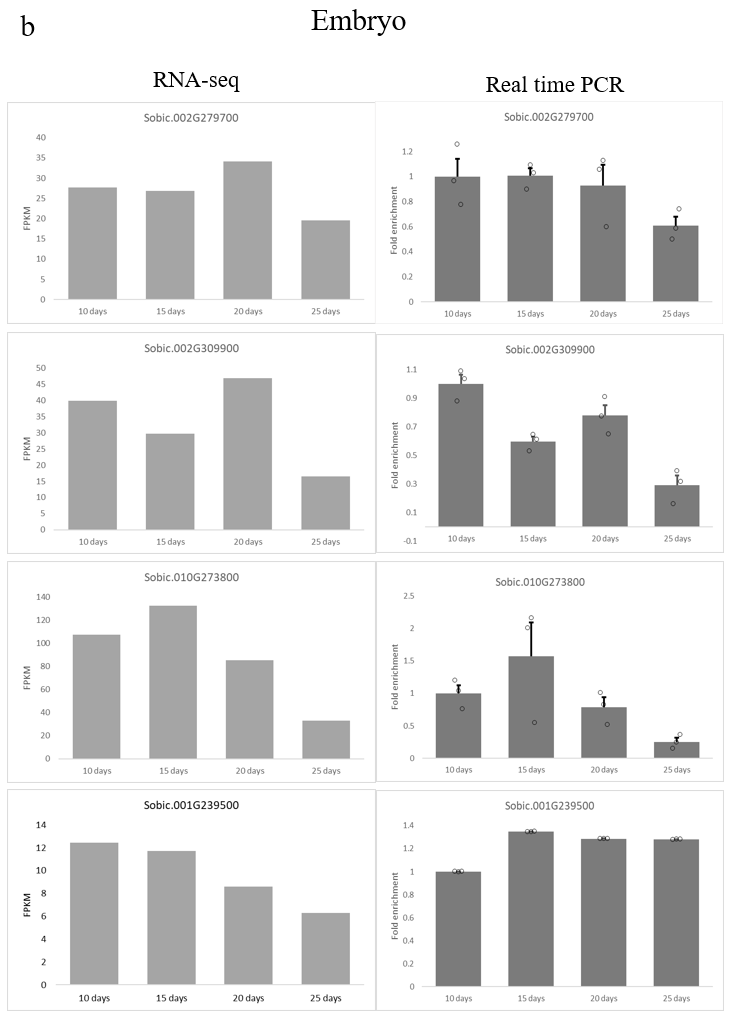


**Supplementary Fig. 5:** Expression patterns of selected genes in early whole seed, endosperm,

and embryo across 5 time points. (a) Representative gene expression levels at 5 days post-anthesis in whole seeds and at 10, 15, 20, and 25 days post-anthesis in endosperm. Left Panels (RNA-seq): Expression levels are quantified as fragments per kilobase of transcript per million mapped reads (FPKM). Right Panels (Real-time PCR): The fold enrichment of gene expression is calculated relative to 5 days whole seeds as control. (b) Representative gene expression levels at 10, 15, 20, and 25 days in embryo tissue. Left Panels (RNA-seq): Expression levels are quantified as FPKM. Right Panels (Real-time PCR): The fold enrichment of gene expression is calculated relative to the 10 days embryo as control. Error bars represent the standard error of the mean (SEM).


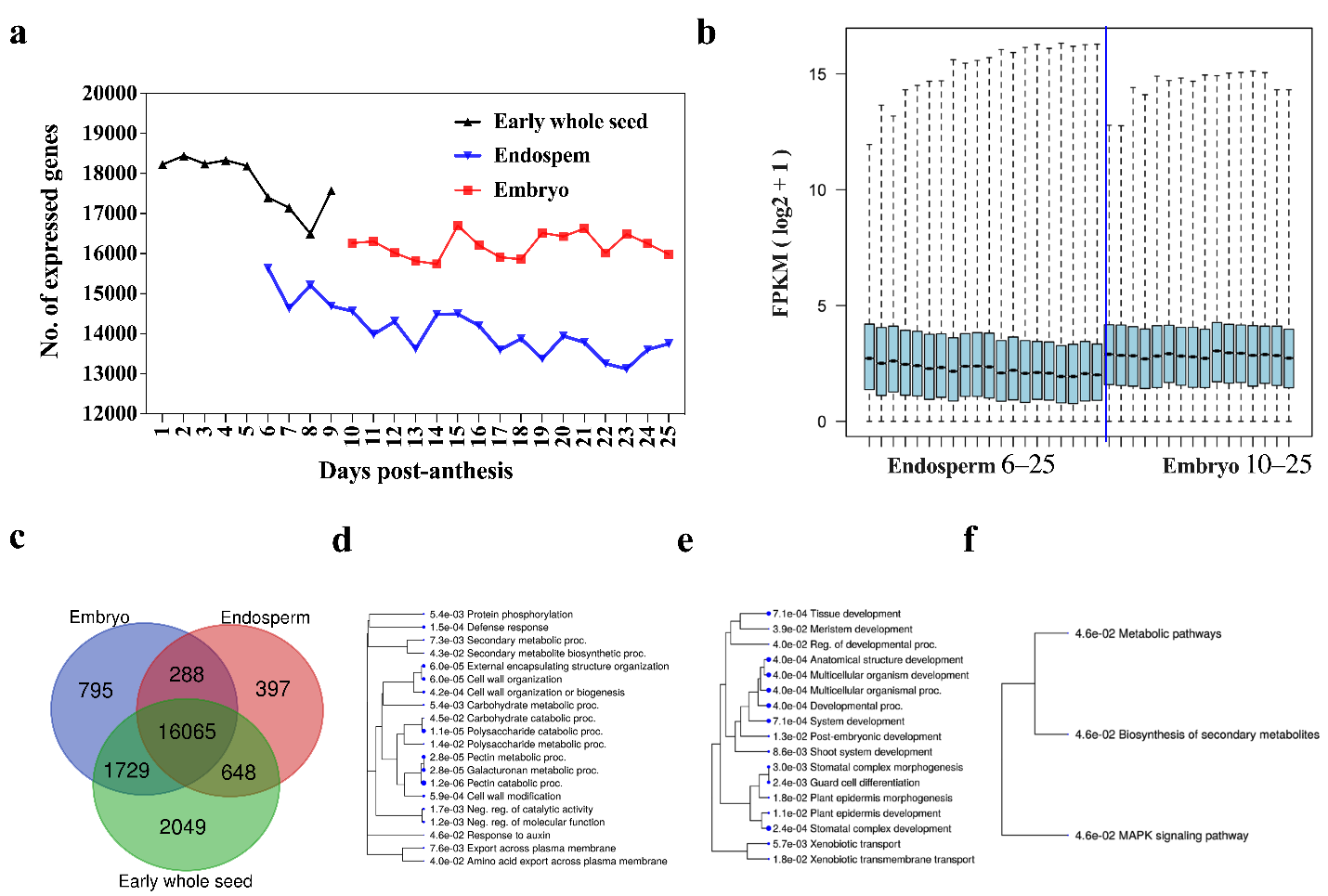


**Supplementary Fig. 6:** (a) Number of genes expressed in early whole seed, endosperm, and embryo across all timepoints. (b) Comparison of expression levels (FPKM) of genes detected in BTx623 embryos and endosperms (c) Venn diagram of the 21,971 genes detected among the embryo, endosperm, and early whole seed. Functional annotation of tissue-specific genes including the early whole seed (d), embryo (e), and endosperm (f).

**
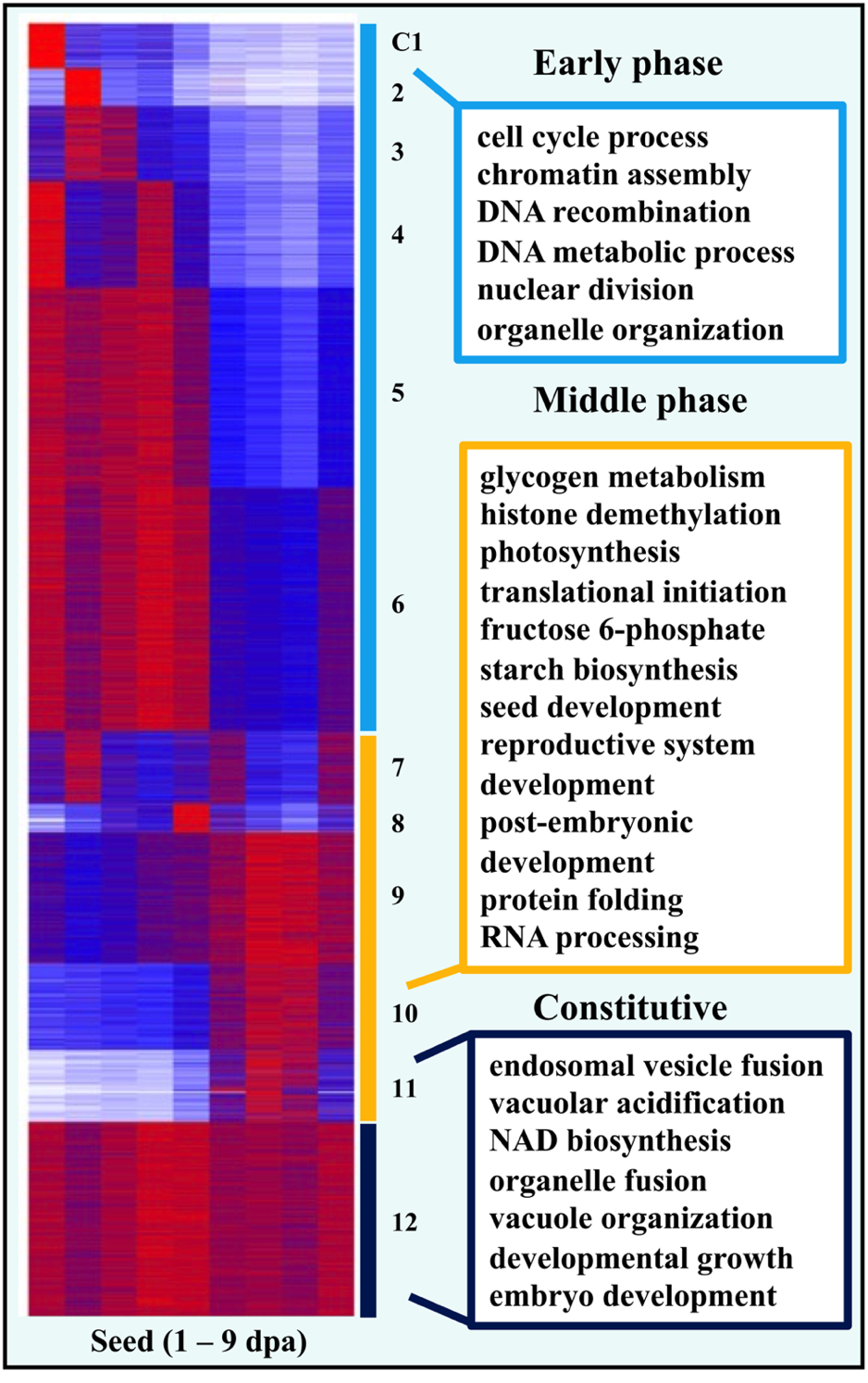
**

**Supplementary Fig. 7**: Expression patterns of co-expression modules for the early whole seed. Functional categories that are enriched within different co-expression clusters for the embryo and endosperm are listed. For each gene, the RPKM value is displayed, normalized in relation to the maximum RPKM value observed for that gene across all time points.

**a**

**
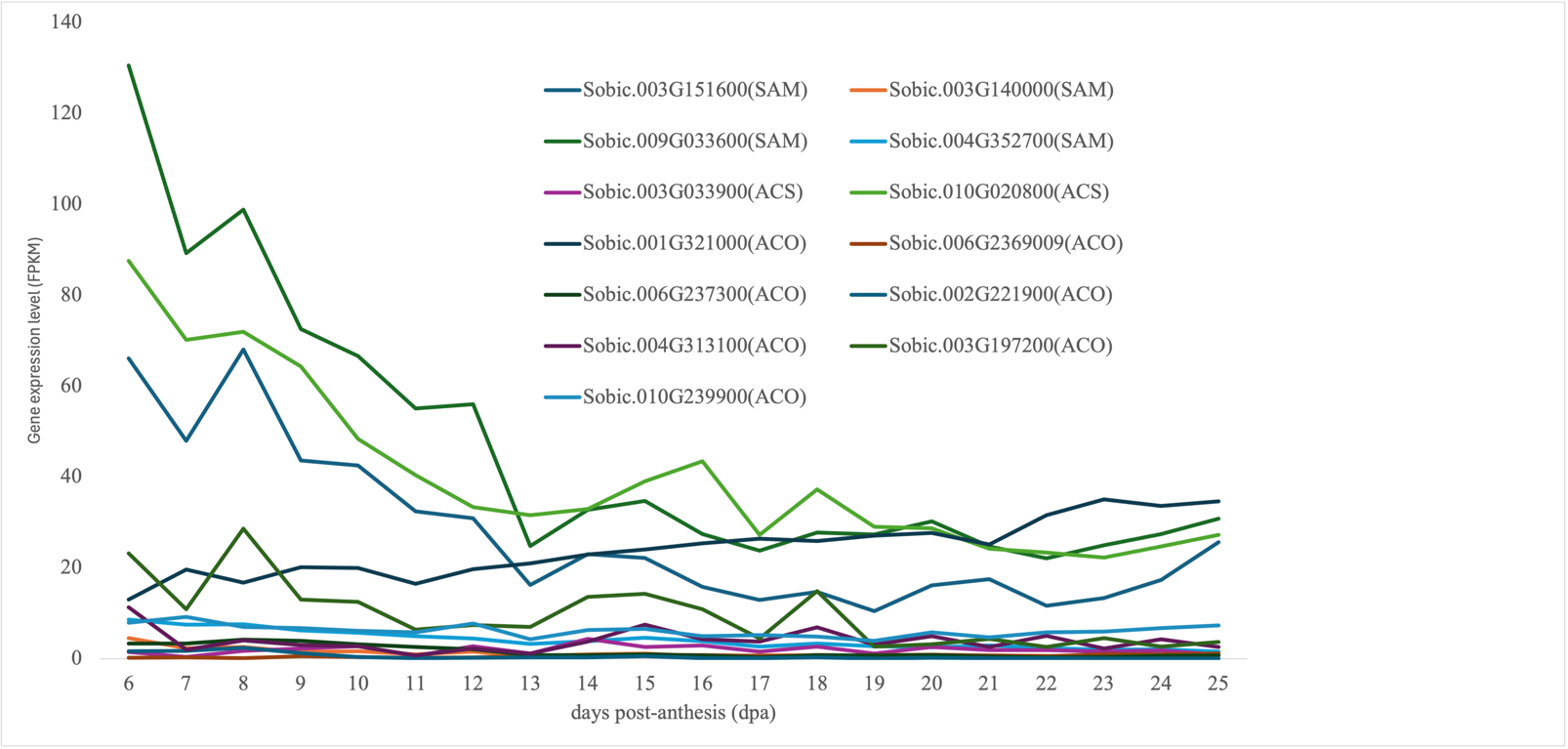
**

**b**


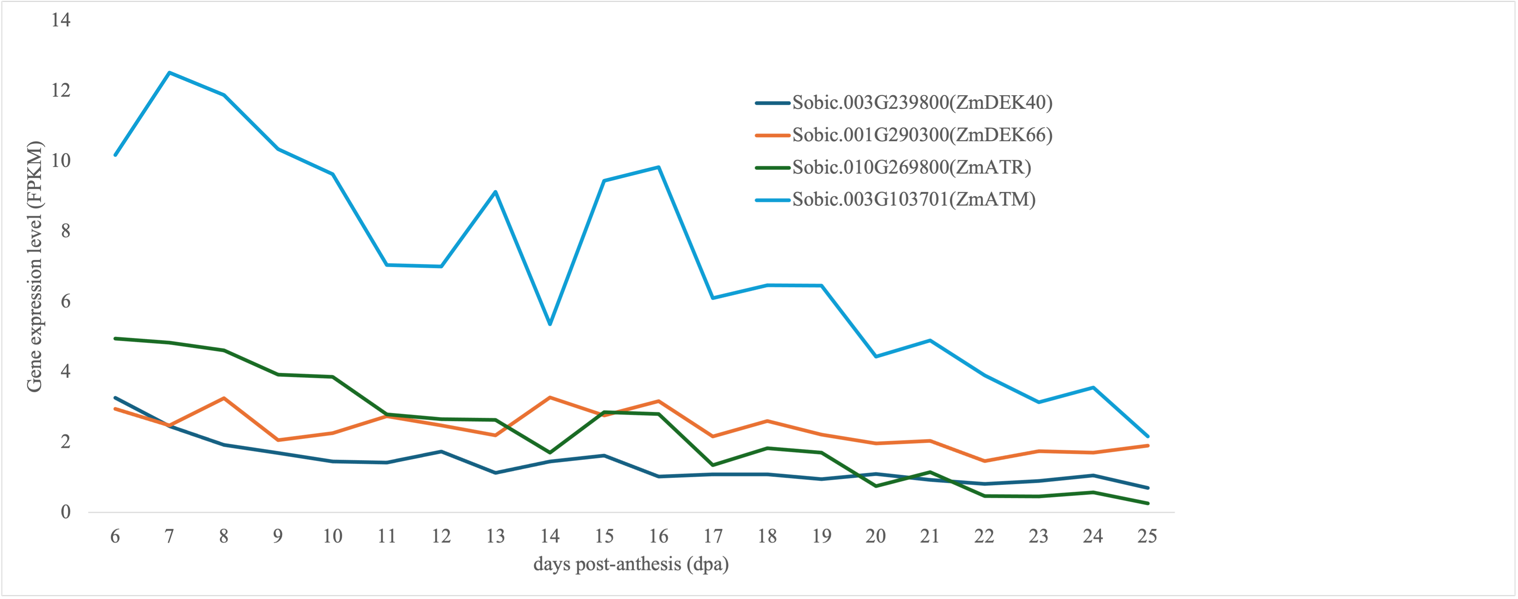


**Supplementary Fig. 8:** (a) The expression level (FPKM) of the ethylene biosynthesis genes in sorghum endosperm development. SAM – methionine adenosyltransferase, ACS - aminocyclopropane-1-carboxylate synthase, ACO - aminocyclopropane-1-carboxylate oxidase; (b) The expression level (FPKM) of the four PCD regulator genes in sorghum endosperm development.


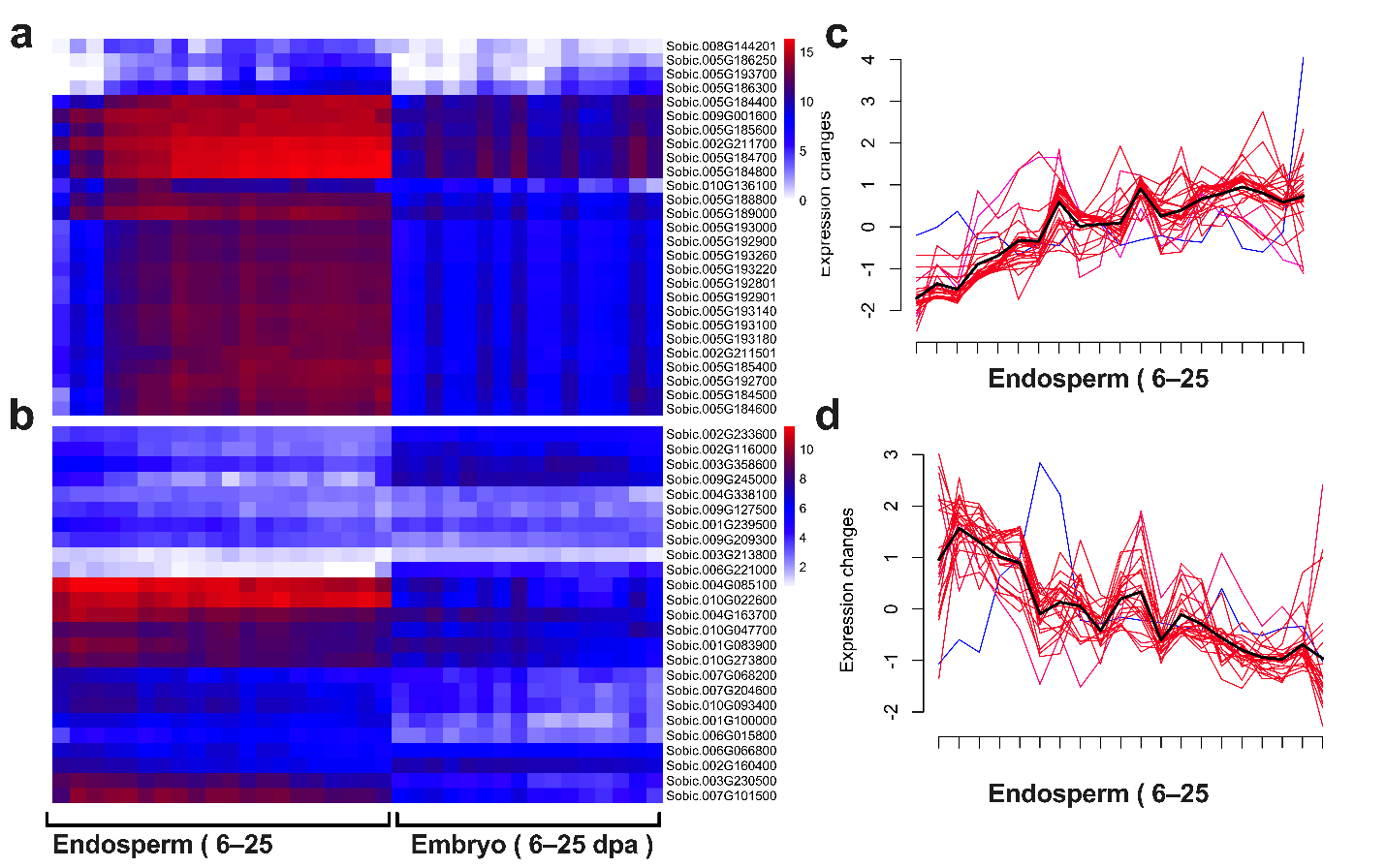


**Supplementary Fig. 9:** (a) Expression profile (Log_2_ FPKM+1) of kafirin genes in the endosperm and embryo. (b) Expression profile (Log_2_ FPKM+1) of starch genes in the embryo and endosperm. (c-d) Expression trend of kafirin (c) and starch (d) genes in the developing endosperm.


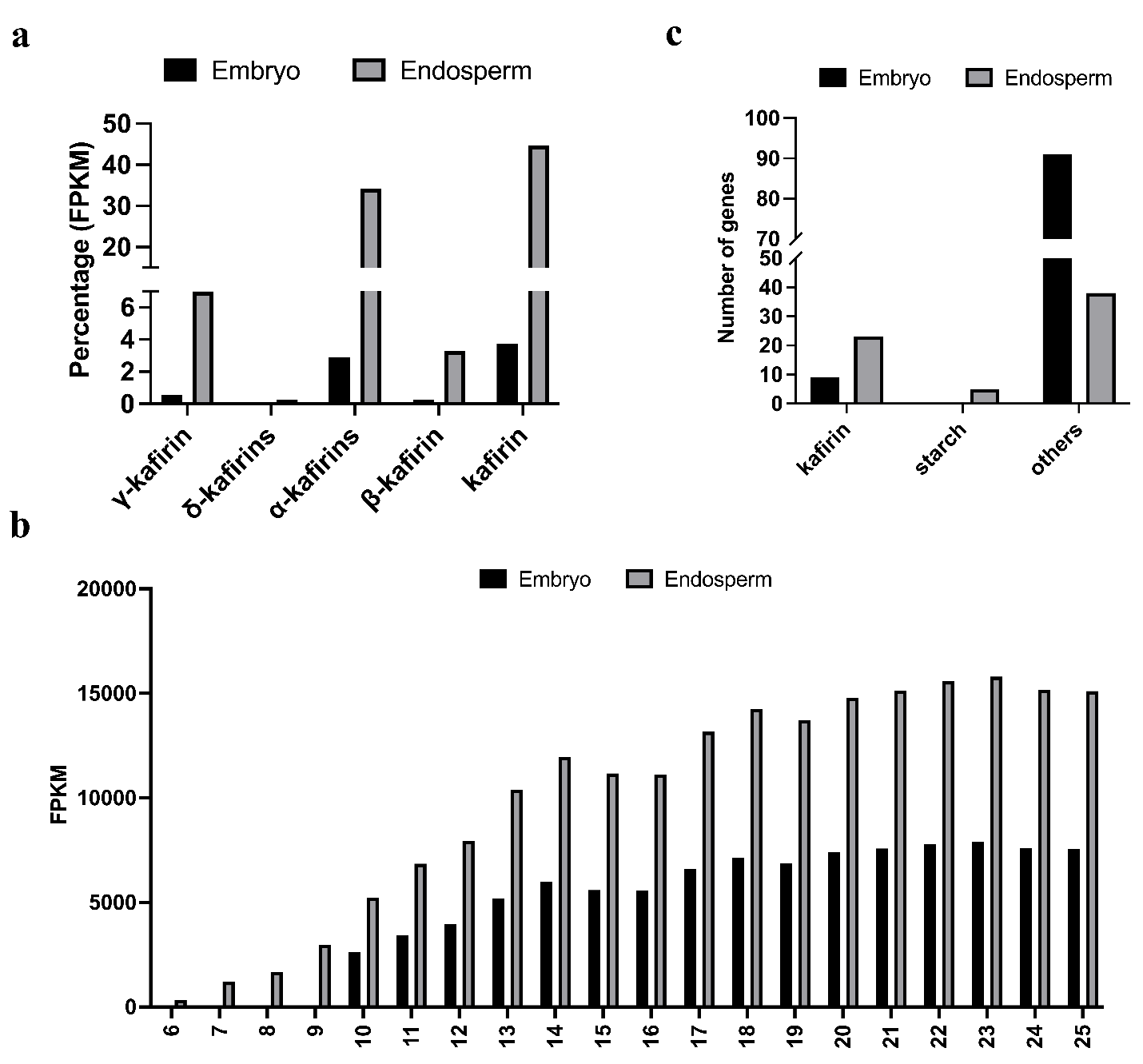


**Supplementary Fig. 10:** Contribution of the various kafirin genes to the total transcriptome. (a) The dynamic transcript levels of different kafirin gene family members in the endosperm and embryo. (b) Expression level (average FPKM of kafirin genes) in endosperm and embryo (c) The distribution of kafirin and starch genes in the 100 most highly expressed genes in the endosperm.

**
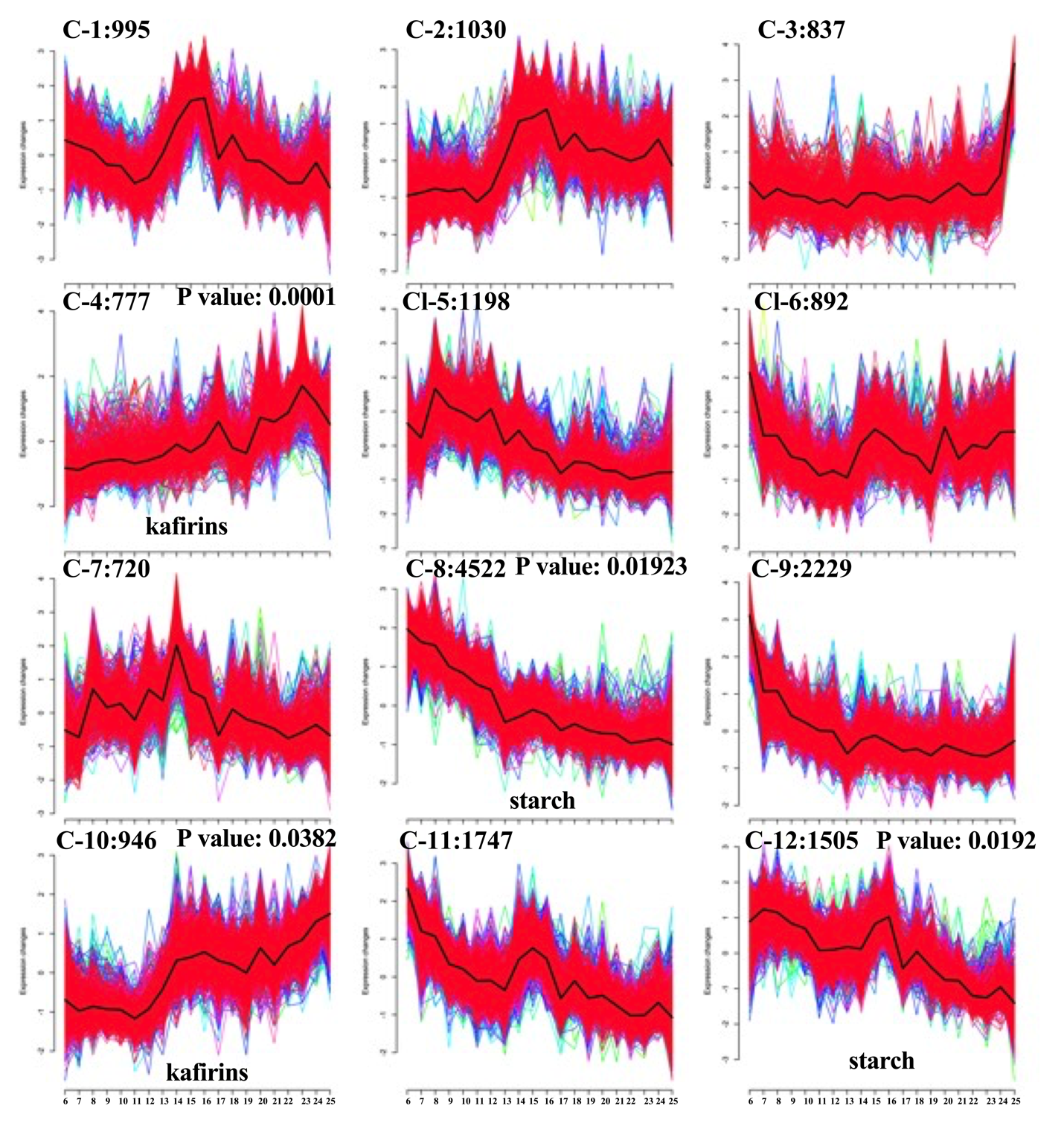
**

**Supplementary Fig. 11:** Fuzzy c-means clustering of the endosperm samples.


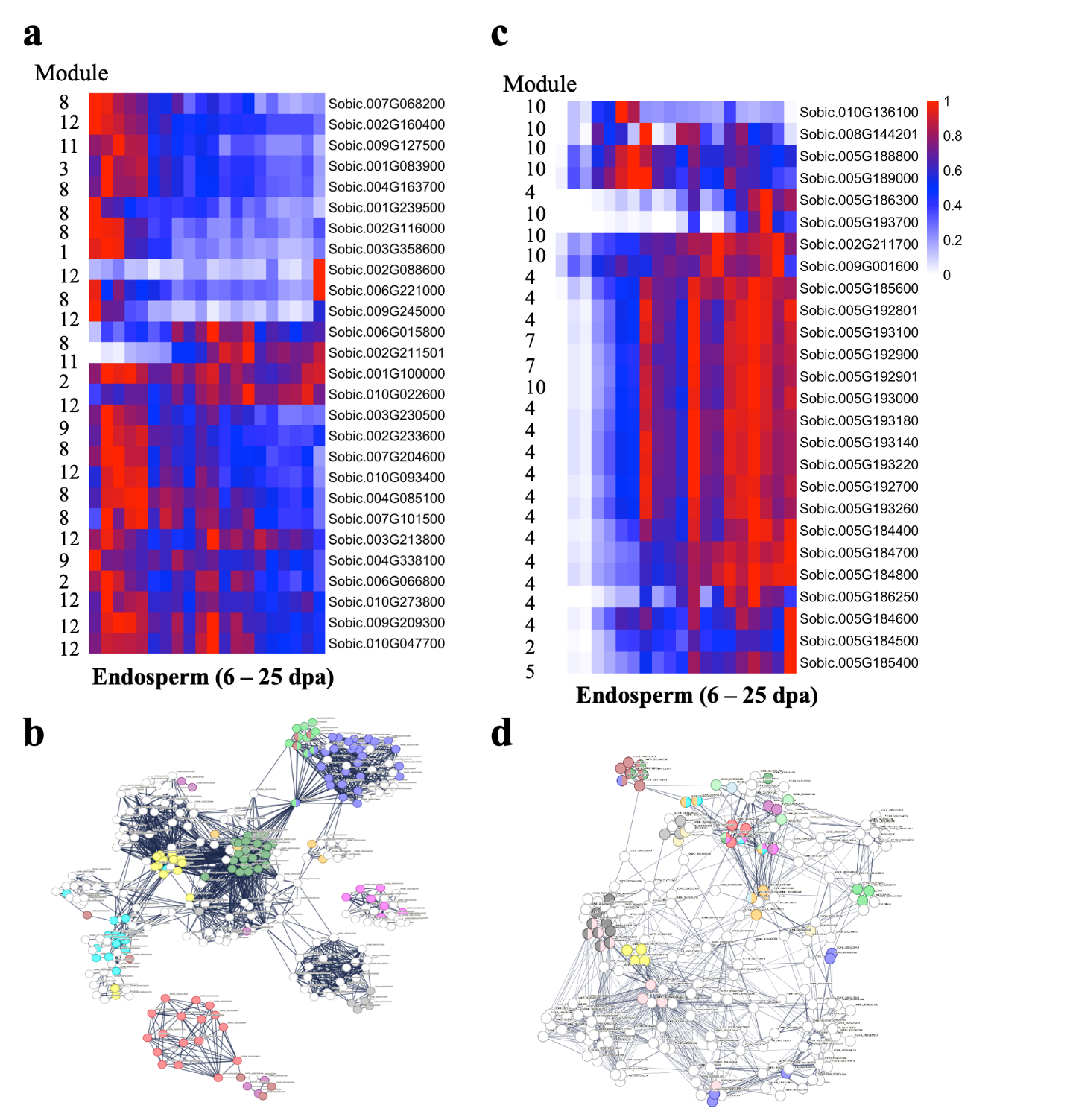


**Supplementary Fig. 12:** (a) The distribution of starch genes in the expression module generated by mfuzz, and its expression in the endosperm. (b) Co-expression network of the genes co-expressed with starch. (c) The distribution of kafirin genes in the expression module generated by mfuzz, and its expression in the endosperm. (d) Co-expression network of the genes co-expressed with kafirin genes.


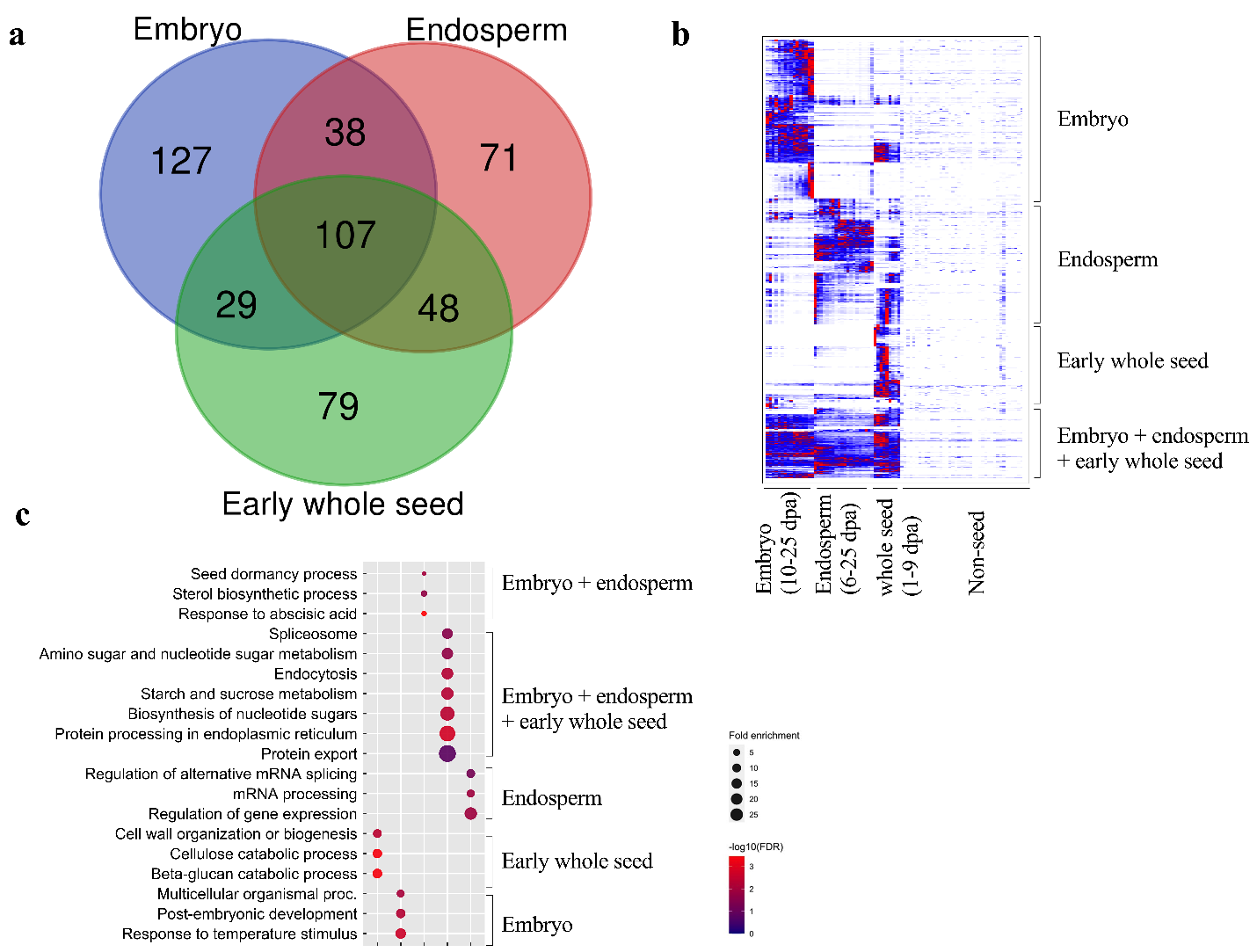


**Supplementary Fig. 13:** Expression patterns of seed-specific genes in seed and non-seed samples. (a) The Venn diagram illustrates the 499 genes detected in the BTx623 embryo, endosperm, and early whole seed. (b) Heat map based on the RPKM value, normalized relative to the maximum RPKM value observed for each gene across all seed and non-seed samples. (c) Tissue-specific genes enriched pathways. The depth of color reflects the adjusted P-value and the circle's area signifies gene counts.
