# Supplementary material for: Transcriptome and metabolome analyses reveal regulatory networks associated with nutrition synthesis in sorghum seeds
Source: Commun Biol. 2024 Jul 10;7:841. doi: 10.1038/s42003-024-06525-7 (PMC11237005; doi:10.1038/s42003-024-06525-7)
Supplement: Supplementary file 3 — Description of Additional Supplementary Files [file 42003_2024_6525_MOESM3_ESM.pdf]

Description of Additional Supplementary Files

File name: Supplementary Data 1

Description: Samples used for transcriptomic analysis in the current study.

File name: Supplementary Data 2

Description: Summary of the metabolome data. The peak intensity value for each biological replicate is shown for all detected metabolites. The file contains information about ion, mode, rt, molecular weight, formula, name, mass, level, annotation such as KEGG and HMDB ID, class and family, and pathway. QC, quality control sample; se, whole seed; rt, retention time. The numbers 5, 10, 15, 20, and 25 along with se indicate days post-anthesis, followed by the number of replicates.

File name: Supplementary Data 3

Description: KEGG annotation and metabolite classification of the identified metabolites.

File name: Supplementary Data 4

Description: Differentially accumulated metabolites among the investigated comparisons (5 vs. 10, 15, 20 and 25 dpa). Up, up-regulated; DOWN, down-regulated.

File name: Supplementary Data 5

Description: Summary of alignment of RNAseq reads to the reference genome: em, embryo; en, endosperm; se, early whole seed.

File name: Supplementary Data 6

Description: Summary of the transcriptome data. The mean value (FPKM) from two biological replicates is shown for each gene in the early whole seed, embryo, and endosperm, along with details such as clustering and tissue specificity. TF, transcription factor; tsGenes, tissue specific genes; hstsGenes, high tissue specific genes; em, embryo; en, endosperm; se, early whole seed. Numbers 10–25 along with em, en, and se indicate days post-anthesis.

File name: Supplementary Data 7

Description: Number of genes and TFs expressed in the BTx623 early whole seed, embryo, endosperm: em, embryo; en, endosperm; se, early whole seed.

File name: Supplementary Data 8

Description: Expression (FPKM) of starch and kafirin genes in the endosperm (en) at different developmental stages.

File name: Supplementary Data 9

Description: List of the 100 most highly expressed genes in the endosperm (en).

File name: Supplementary Data 10

Description: List of the 100 most highly expressed genes in the embryo (em).

File name: Supplementary Data 11

Description: Fuzzy c-means clustering with the Mfuzz v2.42 R package.

File name: Supplementary Data 12

Description: Hub genes associated with starch biosynthesis, ranked by connectivity.

File name: Supplementary Data 13

Description: Hub genes associated with kafirin biosynthesis, ranked by connectivity.

File name: Supplementary Data 14

Description: Oligonucleotides used for RTqPCR.

File name: Supplementary Data 15

Description: The source data behind the graphs in the main figures.
