# Supplementary material for: Transcriptome and metabolome analyses reveal regulatory networks associated with nutrition synthesis in sorghum seeds
Source: Commun Biol. 2024 Jul 10;7:841. doi: 10.1038/s42003-024-06525-7 (PMC11237005; doi:10.1038/s42003-024-06525-7)
Supplement: Supplementary file 19 — Reporting Summary [file 42003_2024_6525_MOESM19_ESM.pdf]

Corresponding author(s): Yinping Jiao

Last updated by author(s): Jun 3, 2024

Reporting Summary

Nature Portfolio wishes to improve the reproducibility of the work that we publish. This form provides structure for consistency and transparency in reporting. For further information on Nature Portfolio policies, see our Editorial Policies and the Editorial Policy Checklist.

Statistics

For all statistical analyses, confirm that the following items are present in the figure legend, table legend, main text, or Methods section.

n/a	Confirmed	
☐	☒	The exact sample size (*n*) for each experimental group/condition, given as a discrete number and unit of measurement
☐	☒	A statement on whether measurements were taken from distinct samples or whether the same sample was measured repeatedly
☐	☒	The statistical test(s) used AND whether they are one- or two-sided<br>*Only common tests should be described solely by name; describe more complex techniques in the Methods section.*
☒	☐	A description of all covariates tested
☐	☒	A description of any assumptions or corrections, such as tests of normality and adjustment for multiple comparisons
☐	☒	A full description of the statistical parameters including central tendency (e.g. means) or other basic estimates (e.g. regression coefficient) AND variation (e.g. standard deviation) or associated estimates of uncertainty (e.g. confidence intervals)
☐	☒	For null hypothesis testing, the test statistic (e.g. F, t, r) with confidence intervals, effect sizes, degrees of freedom and P value noted<br>*Give P values as exact values whenever suitable.*
☒	☐	For Bayesian analysis, information on the choice of priors and Markov chain Monte Carlo settings
☒	☐	For hierarchical and complex designs, identification of the appropriate level for tests and full reporting of outcomes
☐	☒	Estimates of effect sizes (e.g. Cohen's *d*, Pearson's *r*), indicating how they were calculated

Our web collection on statistics for biologists contains articles on many of the points above.

Software and code

Policy information about availability of computer code

Data collection	For transcriptome analysis, developing sorghum seeds were collected with three replicates following successful pollination. Metabolomic analysis involved the collection of five replicates at 5, 10, 20, 25, and 30 dpa. Each replicate comprised 10 uniform panicles within 3-meter plots, each containing 60–70 plants.
Data analysis	1. data underwent QC assessment using FastQC.<br>2. High-quality reads were aligned to the sorghum version 3 reference genome using STAR (2.7.0a).<br>3. The FPKM values representing gene expression levels were calculated using StringTie (v2.2.0).<br>4.R vesion 4.0.5 was used in R studio to plot the data such as PCA were ploted using prcomp anf ggplot2 package. Mfuzz v2.42 R package were used for cluster analysis.<br>5. KEGG and ShinyGO databes were used for gene enrichment analysis. STRING database were used for network analysis.<br>6. The identified metabolites were annotated using the Kyoto Encyclopedia of Genes and Genomes (KEGG) pathway and Human Metabolome Databases (HMDB).The PCA and Partial Least Squares Discriminant Analysis (PLS-DA) were conducted with the metabolomics software MetaboAnalyst93.

For manuscripts utilizing custom algorithms or software that are central to the research but not yet described in published literature, software must be made available to editors and reviewers. We strongly encourage code deposition in a community repository (e.g. GitHub). See the Nature Portfolio guidelines for submitting code & software for further information.

Data

Policy information about availability of data

All manuscripts must include a data availability statement. This statement should provide the following information, where applicable:

- Accession codes, unique identifiers, or web links for publicly available datasets
- A description of any restrictions on data availability
- For clinical datasets or third party data, please ensure that the statement adheres to our policy

The RNA-seq data has been deposited into the Ensembl ArrayExpress collection in BioStudies under Accession number: E-MTAB-13406 (https://www.ebi.ac.uk/biostudies/arrayexpress/studies/E-MTAB-13406?key=b4d13b86-036a-491a-a4da-481faa48c2ff). The metabolomic data, encompassing compound names, formulas, exact Q1 (m/z) values, molecular weights, and peak intensities, are available in the supplementary data.

Human research participants

Policy information about studies involving human research participants and Sex and Gender in Research.

Reporting on sex and gender	N/A
Population characteristics	N/A
Recruitment	N/A
Ethics oversight	N/A

Note that full information on the approval of the study protocol must also be provided in the manuscript.

Field-specific reporting

Please select the one below that is the best fit for your research. If you are not sure, read the appropriate sections before making your selection.

☒ Life sciences ☐ Behavioural & social sciences ☐ Ecological, evolutionary & environmental sciences

For a reference copy of the document with all sections, see nature.com/documents/nr-reporting-summary-flat.pdf

Life sciences study design

All studies must disclose on these points even when the disclosure is negative.

Sample size	Sample size were estimated based on previous studies in the field.
Data exclusions	We have droped one RNA seq sample (5 dpa endosperm). We droped it becasue at 5dpa it was difficult to separate the edosperm from the cell wall (pericarp) of the seed. After sequnsing, we checked the expression of key genes invovled in pericarp formation in was active in this sample, and therefore we droped.
Replication	We have used two biological replicates for RNA-Seq analysis and five replicates for metabolome analysis. we crossed validate each replicates by using key marker genes (tissue and stage specific; data not included in the paper). The expression patterns of these genes indicated high quality of the data. In addition, we have used three biological replicates for RT-qPCR.
Randomization	Each replicate comprised 10 uniform panicles within 3-meter plots, each containing 60–70 plants. For all replicates, plants were randomly chosen among each plots.
Blinding	No blinding was consider in this study.

Reporting for specific materials, systems and methods

We require information from authors about some types of materials, experimental systems and methods used in many studies. Here, indicate whether each material, system or method listed is relevant to your study. If you are not sure if a list item applies to your research, read the appropriate section before selecting a response.

Materials & experimental systems

n/a	Involved in the study
☒	☐ Antibodies
☒	☐ Eukaryotic cell lines
☒	☐ Palaeontology and archaeology
☒	☐ Animals and other organisms
☒	☐ Clinical data
☒	☐ Dual use research of concern

Methods

n/a	Involved in the study
☒	☐ ChIP-seq
☒	☐ Flow cytometry
☒	☐ MRI-based neuroimaging
